# Psychometric properties of the Chinese version of the pros and cons of anorexia nervosa (P-CAN-C) scale: a validation study in patients with anorexia nervosa

**DOI:** 10.1186/s40337-025-01314-x

**Published:** 2025-06-16

**Authors:** Xu Han, Mei-chun Cheung, Xueni Li, Lucy Serpell

**Affiliations:** 1 Department of Social Work, The Chinese University of Hong Kong, Shatin, New Territories, Hong Kong SAR China; 2 Peking University Sixth Hospital, Peking University Institute of Mental Health, NHC Key Laboratory of Mental Health (Peking University), National Clinical Research Center for Mental Disorders (Peking University Sixth Hospital), Beijing, China; 3 University College London, London, UK

**Keywords:** Anorexia nervosa, Pros and cons of anorexia nervosa scale, Scale validation, China

## Abstract

**Background:**

Individuals with anorexia nervosa (AN) often face significant challenges in maintaining motivation for recovery. Understanding the perceived pros and cons associated with the disorder is crucial for promoting recovery. This study aimed to translate, adapt, and validate the Pros and Cons of Anorexia Nervosa Scale (P-CAN) for use with Chinese adults with AN, thereby facilitating a clearer understanding of the motivations and barriers encountered by these patients.

**Methods:**

This study employed a cross-sectional survey design to collect data from 207 Chinese adults with AN (M_age_25.58 and SD 6.011). Content validity was assessed by a panel of professionals. Reliability testing included internal consistency, test-retest reliability, item-total correlation, and correlation analysis between subscales. Principal Component Analysis (PCA) assessed the factor structure, focusing on two components (Pro and Con-AN) and ten subscales—Safe/Structured, Appearance, Fertility/Sexuality, Special, Fitness, Communicate Emotions/Distress for Pro-AN and Trapped, Guilt, Hatred, Stifled for Con-AN as per the original scale. Criterion validity was evaluated using the eating pathology tested by Eating Disorder Examination Questionnaire (EDE-Q) and Body Mass Index (BMI).

**Results:**

The content validity of the overall scale was 0.86. The Pro-AN and Con-AN subscales exhibited strong internal consistency (α = 0.84 and 0.82) and test-retest reliability (*r* = 0.912 and 0.704, *p* < 0.001). Item-Total Correlations exceeded 0.3 for all items except item 2, and there was no significant correlation between the Pro-AN and Con-AN subscales. The PCA results indicated that the Chinese P-CAN retained two components, which are consistent with the original scale. Differences emerged in more granular dimensions that may not be appropriate within the Chinese context. Significant correlations were found between the Pro-AN (*r* = 0.279, *p* < 0.001) and Con-AN (*r* = 0.240, *p* < 0.001) subscales and eating pathology and Con-AN was significantly correlated with BMI (*r* = -0.214, *p* < 0.01).

**Conclusions:**

The P-CAN has shown promising psychometric properties among Chinese patients with AN. In terms of dimensionality, the Chinese context aligns closely with the original scale’s binary division into Pro-AN and Con-AN. However, the further differentiation into ten dimensions, may not be culturally appropriate for the Chinese context.

**Supplementary Information:**

The online version contains supplementary material available at 10.1186/s40337-025-01314-x.

## Abstract (plain language summary)

This study aimed to translate and culturally adapt the Pros and Cons of Anorexia Nervosa Scale (P-CAN) for Chinese adults with anorexia nervosa (AN). The adapted scale is designed to assess motivations and barriers among Chinese individuals with AN in the recovery process, providing insights for clinical practice.

The study involved 207 Chinese adults with AN, who completed the translated scale. The scale was tested for accuracy (content validity), consistency (reliability), and its ability to measure what it’s supposed to measure (validity). The results showed that the Chinese version of the P-CAN is reliable and valid for use with Chinese adults with AN.

The scale divides into two main parts: Pro-AN (reasons patients might see benefits in their disorder) and Con-AN (reasons they might see drawbacks). These two parts showed strong consistency and reliability. However, while the overall structure of the scale matched the original version, some of the more detailed subcategories (like “Safe/Structured” or “Trapped”) might not fit as well in the Chinese cultural context. The study also found that both the Pro-AN and Con-AN parts of the scale were linked to eating disorder symptoms, and the Con-AN part was also linked to body weight (BMI).

In conclusion, the Chinese version of the P-CAN is a useful tool for understanding the motivations and challenges of Chinese adults with AN. While the overall structure works well, some of the finer details might need adjustment to better fit the Chinese cultural context. This tool can help clinicians and researchers better support patients in their recovery.

**Supplementary Information:**

The online version contains supplementary material available at 10.1186/s40337-025-01314-x.

## Background

Anorexia nervosa (AN) is a mental disorder characterised by a persistent restriction of energy intake, leading to significantly low body weight, intense fear of gaining weight or becoming fat, and a disturbance in how one’s body weight or shape is experienced [1]. AN has a high mortality rate associated with significant physical and psychological morbidity [2]. According to a recent national survey, China has more than 1.31 million people affected by AN, representing a significant increase [3, 4] compared to the global situation. Notably, the incidence of AN in China has exhibited a continuous upward trend, while worldwide incidence rates have remained stable [4]. Despite the development of effective treatments, many patients with AN struggle to recover, and a better understanding the factors contributing to the disorder’s maintenance may contribute to adaptations to treatment to benefit more sufferers [5].

Motivation is an important factor in the recovery from AN [6–8], and plays a significant role in treatment outcomes; higher motivation prior to treatment is associated with more favorable results [9]. Patients with AN often have ambivalent feelings about recovery, and their motivation to change can be influenced by various factors, including individual characteristics, co-morbid psychopathology, lack of treatment autonomy and relationships with others [7]. Patients’ perceptions of the disorder—including their views on their own illness, related problems, hospitalization, healthcare personnel, fellow patients, and the clinical environment—are important influencing factors closely linked to their motivation as well [10]. In the context of AN, the valued nature of AN, often abbreviated as Pro-AN, refers to the aspects of the disorder that are perceived as beneficial or desirable [11]. These perceptions may include identification with thinness, the pursuit of a sense of control, and the enhancement of self-worth [12].These perceptions are considered critical in sustaining the disorder and have been identified as a key component of the cognitive-interpersonal maintenance model of AN [11], which may contribute to maintaining the disorder by reinforcing the patient’s desire to continue restricting their food intake and reducing their motivation to change [5], often leading patients to exhibit resistance and ambivalence when confronted with treatment [12]. Conversely, negative beliefs and consequences about AN (Con-AN) can serve as a motivating factor for change and recovery [12]. Patients’ motivation is often complex, influenced by both positive and negative perceptions (Pro-AN & Con-AN) [12]. In the therapeutic process, identifying and exploring these beliefs can enhance patients’ motivation and facilitate recovery [13].

To better understand the pros and cons of AN from the perspective of patients themselves, and use it to predict the maintenance of AN, researchers have developed the Pros and Cons of Anorexia Nervosa (P-CAN) scale, which measures the pros and cons of AN beliefs [12, 14]. The P-CAN scale originated from patients’ subjective experiences, which were qualitatively coded and classified in the original study [13]. Through qualitative research methods, these experiences were systematically analyzed and categorized [13]. The development of the P-CAN scale began with an initial classification into two primary dimensions: positive (Pro) and negative (Con) aspects of AN. This broad categorization provided a general understanding of patients’ experiences [12]. Subsequent principal components analysis (PCA) identified ten distinct subscales—six for pros (Safe/Structured, Appearance, Fertility/Sexuality, Special, Fitness, Communicate Emotions/Distress) and four for cons (Trapped, Guilt, Hatred, Stifled)—reflecting a more nuanced structure [12]. This shift from a two-dimensional to a ten-dimensional framework enhances the scale’s depth, allowing for a comprehensive understanding of the complex attitudes towards AN, thereby increasing its utility in clinical and research contexts [12].

The results of the original version indicate P-CAN exhibits robust psychometric properties, establishing it as a valuable tool for assessing attitudes towards AN and offering insights into the factors that contribute to the maintenance of the disorder [12]. Pro-AN are significant indicators of the factors that may perpetuate AN [13] which is very useful in clinical setting. However, to the best of our knowledge, no published versions of the questionnaire other than the English version could be retrieved.

Considering the cultural differences in experiences of mental disorders—such as help-seeking behaviors, stigma, coping styles, social support, and the meanings patients attribute to their illnesses [15], it is possible that the P-CAN may shows difference in patients’ perception about AN between Western and other cultural contexts. For example, Chinese culture places strong emphasis on the principle of “body and hair belonged to one’s parents,” leading to a more cautious attitude towards weight loss among Chinese individuals [16, 17]. Simultaneously, weight control can be interpreted as a form of defiance or criticism towards parents, which may influence patients’ perceptions of AN [18, 19]. It is essential to validate the use of the P-CAN scale before using it in different cultural contexts. Conducting a validation of the Chinese version of the scale is necessary to ensure its appropriateness and reliability in capturing the intended constructs across diverse cultural settings.

### Current study

The current study aimed to translate the P-CAN scale into Chinese (P-CAN-C) and validate it among Chinese adults with AN.

## Methods

### Participants

Participants were recruited from mainland China through staff at specialized hospitals and online platforms. Psychiatrists and psychotherapists reached out to patients who had previously received or were currently undergoing treatment through meetings, personal online platforms, and the instant messaging application WeChat. Researchers also disseminated recruitment information for the study through WeChat public platforms in Mainland China that have a significant reach in the promotion of eating disorders. Those included in this study were diagnosed with AN or Eating Disorder Not Otherwise Specified– Anorexia Nervosa (EDNOS-AN) based on the Diagnostic and Statistical Manual of Mental Disorders, Fifth Edition Text Revision (DSM-5TR) [1] by clinicians, aged 18 years or older, and able to read and write Chinese. Patients with severe comorbidities at the time of the survey (e.g., learning disabilities or psychosis) were excluded. This study has been approved by Survey and Behavioral Research Ethics of The Chinese University of Hong Kong. The ethical approval reference number is No. SBRE-23–0250 A. All participants provided informed consent, either online or in written form. Participation was voluntary, and participants were offered a coffee voucher valued at 20 RMB as an incentive.

### Sampling method

A convenience sampling method was used in the current study. This approach was taken because nonprobability samples can be created when the sampling units appear representative and can be conveniently accessed [20, 21]. Consecutive enrollment was also used, meaning that all eligible cases who agree to participate were included in the study.

### Measurements

#### Demographic variables and treatment status

Demographic variables of participants, including gender, age, educational and occupational status, as well as their treatment status related to AN, encompassing diagnosis and treatment options, were assessed.

#### Pros and cons of anorexia nervosa (P-CAN) scale

was utilized to assess the positive and negative aspects of AN. The Pro scales encompass six subscales: Safe/Structured, Appearance, Fertility/Sexuality, Special, Fitness, and Communication, with a total of 30 items distributed across these subscales. In contrast, the Con scales consist of four subscales: Trapped, Guilt, Hatred, and Stifled, with a total of 20 items [14]. The original English version of the P-CAN demonstrates good reliability and validity [12], with Cronbach’s alpha values ranging from 0.68 to 0.89 [12].

The P-CAN scale was translated into Chinese utilizing a standardized forward-backward translation procedure, with the translated version provided in the Appendix. A panel of experts then reviewed the translated scale to ensure its cultural and linguistic equivalence. The panel consisted of a psychiatrist with over 20 years of experience working in the field of AN (MD), a licensed social worker who is also a PhD student, a psychotherapist who is also a PhD student, a recovered eating disorder patient currently pursuing a master’s degree in psychology (with overseas study experience and formal training in English language and literature), and a professional proofreader. Following the criterion test, a small-scale pilot examination was conducted to verify the comprehensibility of the translation and to gather feedback from participants.

#### Criterion-related assessment tool: motivational scale

was used to test participants’ motivation, which has been shown to correlate with both Pro-AN and Con-AN. Pro-AN was associated with lower motivation, while Con-AN was linked to higher motivation [13]. The motivational scale was assessed using an analog scale that evaluates, through four distinct types of questions, the subjective desire of patients with eating disorders to receive treatment, as referenced from Bussolotti, Fernández-Aranda [22]. These questions included: (1) To what extent do you perceive the severity of your eating issues? (2) How willing are you to accept treatment for your eating problems? (3) To what degree do you believe that receiving treatment is necessary? and (4) To what extent do you feel that your eating problems interfere with your normal daily life [22]? The scale ranges from 0 to 8, allowing for a nuanced assessment of patients’ motivation levels [22]. In this study, the Cronbach’s alpha coefficient for the motivational scale was 0.73.

#### Indicators of clinical sensitivity

The clinical outcomes of AN encompass two components: physical indicators, commonly represented by Body Mass Index (BMI) [1], and eating pathology, which was assessed using the Eating Disorder Examination Questionnaire (EDE-Q) in this study. The EDE-Q is designed to evaluate the range, frequency, and severity of behaviors associated with a diagnosis of an eating disorder, which is a widely utilized measurement tool that demonstrates strong psychometric properties [23, 24], thereby ensuring clinical sensitivity in assessment. It is categorized into four subscales: Restraint, Eating Concern, Shape Concern, and Weight Concern, along with an overall global score, where a higher score indicates more significant eating difficulties [23, 24]. The Cronbach’s alpha coefficient for the scale in this study is 0.92.

### Data analysis

Descriptive statistics summarized the participants’ demographic and clinical characteristics. Cronbach’s alpha assessed the internal consistency of the Chinese version of the P-CAN Scale. Spearman correlations and PCA evaluated validity. All analyses were conducted in SPSS [25].

### Sample size estimation

Given the relatively small overall population of individuals with AN (prevalence below 0.1% in China [3]), obtaining samples for research purposes posed significant challenges. Therefore, in our statistical approach, we opted for computational methods suitable for small sample sizes, such as bootstrap PCA, to ensure the robustness of the analysis [26]. Wu [27] recommend a sample size of at least 50 to 60 for estimating standard errors. In the case of confidence intervals larger sample sizes (e.g., at least 100) may be needed.

## Results

### Content validation

The content validity of the Chinese version was assessed by three professionals and two patients before the formal administration, who evaluated the scale based on its relevance, comprehensiveness, and representativeness. Each item was rated on a five-point scale, focusing specifically on the relevance of the items about the Pro-AN and Con-AN respectively (details in Table 1). Based on the Item Content Validity Index (ICVI), the CVI for the scale was determined to be 0.86. The Item Content Validity Index (ICVI) for items 25 (Because I have anorexia, I don’t have to worry about getting pregnant) and 29 (Anorexia helps me control my emotions) were found to be below 0.8. Feedback from the professionals and patients indicated that item 29 should be classified as reflecting the positive aspect of the disorder, while item 25 could not be easily categorized as either positive or negative, as individual needs and perceptions may vary significantly. Some raters believe that the association between pregnancy and anorexia is not as closely related as described in other contexts. For item 1 and item 8, a portion of the raters expressed concerns that the language used was not sufficiently aligned with standard Chinese. In response to this feedback, language modifications were made before the formal administration of the assessment. Specifically, the term “fitter” was translated as “healthier” rather than “stronger,” “consistent” was rendered as “uniform” instead of “persistent,” and “hate” was translated as “dislike” rather than “detest.” The dimensional categorization of item 29 remained open and was to be determined based on subsequent analyses. Regarding item 25, given that fertility constitutes a relevant concern for some patients, it remained included within the scale, with its dimensional affiliation determined based on subsequent data. Subsequently, a small-scale pilot examination involving 25 patients with AN was conducted. Participants provided feedback on the questionnaire items and any of these where they felt the meaning was unclear either in person or through instant messaging tools. The researchers made corresponding modifications based on this feedback and reported the findings to the translation team for further discussion to agree the final version of the scale.

**Table 1:** CVI scores for each item

Pro-AN Item	I-CVI	Con-AN Item	I-CVI
P-CAN1	0.6	P-CAN3	1
P-CAN2	0.8	P-CAN5	1
P-CAN4	1	P-CAN7	1
P-CAN6	1	P-CAN9	0.8
P-CAN8	0.4	P-CAN12	0.8
P-CAN10	0.8	P-CAN14	1
P-CAN11	1	P-CAN18	1
P-CAN13	1	P-CAN21	1
P-CAN15	1	P-CAN27	1
P-CAN16	1	P-CAN29	0.4
P-CAN17	1	P-CAN30	1
P-CAN19	1	P-CAN31	0.8
P-CAN20	0.8	P-CAN32	1
P-CAN22	1	P-CAN33	0.8
P-CAN23	0.8	P-CAN38	1
P-CAN24	1	P-CAN40	1
P-CAN25	0.4	P-CAN43	1
P-CAN26	1	P-CAN47	1
P-CAN28	1	P-CAN49	1
P-CAN29	1	P-CAN50	1
P-CAN34	0.8	-	-
P-CAN35	0.8	-	-
P-CAN36	1	-	-
P-CAN37	1	-	-
P-CAN39	1	-	-
P-CAN41	1	-	-
P-CAN42	1	-	-
P-CAN44	1	-	-
P-CAN45	0.8	-	-
P-CAN46	0.6	-	-
P-CAN48	0.8	-	-

Note: Item Content Validity Index (ICVI). P-CAN n, represents the nth item of the Pros and Cons of Anorexia Nervosa Scale

### Demographic characteristics

The study administered a total of 238 questionnaires, of which 207 were considered valid. Among the valid responses, the participants comprised 12 males and 195 females. Additionally, the sample included individuals diagnosed with AN Purging subtype (*n* = 91), AN Restricting subtype (*n* = 88), and Eating Disorder Not Otherwise Specified (*n* = 28). The average age of the participants is 25.58 years, with a standard deviation of 6.011 years. The mean current BMI was 18.49 kg/m² with a standard deviation of 3.698 kg/m². The lowest recorded BMI was 14.40 kg/m² with a standard deviation of 3.047 kg/m². The duration of the participants’ illness had a mean of 76.44 months with a standard deviation of 48.972 months. Among the participants, 30.0% had received psychiatric treatment, 38.2% had undergone psychological therapy, and 13.5% had engaged in nutritional therapy within the past six months. Participants’ sociodemographic characteristics and treatment status over the past six months are presented in Table 2.

**Table 2:** Participants’ sociodemographic characteristics

Variable	Option	Count/Mean	Percentage/SD
Gender	Female	195	94.2%
	Male	12	5.8%
Duration of illness (months)	-	76.44	48.972
Age	-	25.58	6.011
Lowest BMI (kg/m^2^)	-	14.40	3.047
BMI (kg/m^2^)	-	18.49	3.698
Diagnosis	ANP	91	44.0%
	ANR	88	42.5%
	EDNOS	28	13.5%
Level of education	Undergraduate and above	188	90.8%
	Middle school	17	8.2%
Occupational status	Not engaged	35	16.9%
	At work	73	35.3%
	In school	83	40.1%
	Others	11	5.3%
Psychiatric treatment	No	145	70.0%
	Yes	62	30.0%
Psychotherapy and counseling	No	128	61.8%
	Yes	79	38.2%
Nutritional therapy	No	179	86.5%
	Yes	28	13.5%

### Reliability

#### Internal consistency

In this study, the Cronbach’s alpha coefficient for P-CAN was 0.90, the Pro-AN subscale was 0.84, and for the Con-AN subscale was 0.82. The Cronbach’s alpha coefficients for the subscales are reported as: Safe/Structured at 0.86, Appearance at 0.81, Fertility/Sexuality at 0.85, Special at 0.79, Fitness at 0.71, Communicate Emotions/Distress at 0.72, Trapped at 0.60, Guilt at 0.84, Hatred at 0.79, and Stifled at 0.63.

#### Test-retest reliability

The test-retest reliability was assessed by conducting a retest on 30 participants with an interval of one to three weeks. The results indicated that the reliability coefficient for Pro-AN was 0.912, while for Con-AN, it was 0.704 (*p* < 0.001).

#### Item-total correlation

The item-total correlation analysis revealed that, with the exception of item 2, all other items demonstrated a correlation coefficient exceeding 0.3 in at least one dimension. The correlations of items 40 and 45 with both dimensions exceed 0.3; however, the correlation in one dimension is significantly higher than in the other (details in Table 3).

**Table 3:** Item-Total correlation matrix for Pro-AN and Con-AN subscales

Item	Pro-AN	Con-AN	Item	Pro-AN	Con-AN
P-CAN1	0.513***	-0.196**	P-CAN26	0.441***	0.169*
P-CAN2	0.210**	0.215**	P-CAN27	-0.220**	0.677***
P-CAN3	-0.221**	0.643***	P-CAN28	0.538***	-0.090
P-CAN4	0.508***	-0.135	P-CAN29	0.573***	0.013
P-CAN5	-0.242***	0.565***	P-CAN30	-0.004	0.596***
P-CAN6	0.664***	-0.123	P-CAN31	-0.156*	0.612***
P-CAN7	-0.110	0.616***	P-CAN32	-0.198**	0.702***
P-CAN8	0.531***	0.074	P-CAN33	-0.023	0.533***
P-CAN9	-0.039	0.652***	P-CAN34	0.616***	-0.117
P-CAN10	0.538***	-0.312***	P-CAN35	0.697***	-0.100
P-CAN11	0.658***	-0.215**	P-CAN36	0.633***	-0.132
P-CAN12	-0.144*	0.550***	P-CAN37	0.631***	-0.117
P-CAN13	0.641***	-0.061	P-CAN38	-0.062	0.554***
P-CAN14	0.023	0.547***	P-CAN39	0.624***	-0.306***
P-CAN15	0.629***	-0.092	P-CAN40	-0.317***	0.692***
P-CAN16	0.596***	-0.199**	P-CAN41	0.694***	-0.185**
P-CAN17	0.551***	0.127	P-CAN42	0.544***	0.095
P-CAN18	0.201**	0.420***	P-CAN43	-0.132	0.612***
P-CAN19	0.694***	-0.093	P-CAN44	0.622***	-0.145*
P-CAN20	0.690***	-0.075	P-CAN45	0.652***	-0.324***
P-CAN21	-0.101	0.549***	P-CAN46	0.684***	-0.160*
P-CAN22	0.335***	0.232***	P-CAN47	-0.104	0.676***
P-CAN23	0.639***	0.084	P-CAN48	0.544***	-0.262***
P-CAN24	0.416***	0.165*	P-CAN49	-0.106	0.579***
P-CAN25	0.583***	-0.093	P-CAN50	-0.143*	0.678***

Note: * *p* < 0.05 ** *p* < 0.01 *** *p* < 0.001. P-CAN n, represents the nth item of the Pros and Cons of Anorexia Nervosa Scale

#### Correlation analysis

The analysis reveals that there is no significant correlation between Pro-AN and Con-AN. However, when examining the sub-dimensions, it is observed that the correlations for the dimensions of Safe/structured, Appearance, and Fitness exceed 0.6. Additionally, the correlations for the dimensions of Stifled and Hatred surpass 0.6 (details in Table 4).

**Table 4:** P-CAN subscales correlations

	Safe Structured	Appearance	Fertility/ Sexuality	Special	Fitness	Communicate Emotions/ Distress	Trapped	Guilt	Hatred	Stifled	Pro-AN	Con-AN
Safe Structured	1.000	0.640***	0.515***	0.631***	0.574***	0.446***	-0.075	-0.136	-0.217**	0.046	0.843***	-0.139*
Appearance	0.640***	1.000	0.363***	0.582***	0.591***	0.256***	-0.086	-0.191**	-0.171*	0.037	0.778***	-0.144*
Fertility Sexuality	0.515***	0.363***	1.000	0.447***	0.587***	0.249***	-0.061	-0.157*	-0.201**	-0.031	0.709***	-0.142*
Special	0.631***	0.582***	0.447***	1.000	0.589***	0.338***	0.076	-0.012	-0.076	0.069	0.794***	-0.000
Fitness	0.574***	0.591***	0.587***	0.589***	1.000	0.186**	-0.149*	-0.209**	-0.278***	-0.075	0.778***	-0.222**
Communicate Emotions Distress	0.446***	0.256***	0.249***	0.338***	0.186**	1.000	0.223**	0.184**	0.206**	0.281***	0.529***	0.254***
Trapped	-0.075	-0.086	-0.061	0.076	-0.149*	0.223**	1.000	0.429***	0.677***	0.526***	-0.026	0.804***
Guilt	-0.136	-0.191**	-0.157*	-0.012	-0.209**	0.184**	0.429***	1.000	0.487***	0.407***	-0.116	0.758***
Hatred	-0.217**	-0.171*	-0.201**	-0.076	-0.278***	0.206**	0.677***	0.487***	1.000	0.518***	-0.173*	0.811***
Stifled	0.046	0.037	-0.031	0.069	-0.075	0.281***	0.526***	0.407***	0.518***	1.000	0.075	0.749***
Pro-AN	0.843***	0.778***	0.709***	0.794***	0.778***	0.529***	-0.026	-0.116	-0.173*	0.075	1.000	-0.090
Con-AN	-0.139*	-0.144*	-0.142*	-0.000	-0.222**	0.254***	0.804***	0.758***	0.811***	0.749***	-0.090	1.000

Note: * *p* < 0.05 ** *p* < 0.01 *** *p* < 0.001. Pro-AN represents Pro scales of Pros and Cons of Anorexia Nervosa Scale; Con-AN represents Con scales of Pros and Cons of Anorexia Nervosa Scale

### Dimensionality

In this study, consistent with previous methodology [12], a PCA was conducted with setting fixed principal components (PCs) according to the established framework. This first PCA was specifically focused on two PCs (Pro and Con-AN). The Kaiser-Meyer-Olkin Measure of Sampling Adequacy (KMO) value of 0.870, in conjunction with a highly significant Bartlett’s Test of Sphericity (χ^2^ = 5493.945, df = 1225, *p* < 0.001), demonstrates substantial intercorrelation among the variables, thereby confirming the appropriateness for PCA. The results of the PCA revealed that PC1 accounts for 21.479% of the total variance, while PC2 explains an additional 15.462%, culminating in a cumulative variance explanation of 36.940%. It was found that PC 1 comprised 31 items, while PC 2 included 19 items. These components correspond to the two subscales, labeled Pro-AN and Con-AN, respectively. The items included in the two principal components are almost identical, except for item 29 (which is part of the Con-AN subscale in the original version but is classified as Pro-AN in the Chinese version) (details in Table 5).

**Table 5:** Rotated component matrix for PCA results of the P-CAN scale: two component solutions

Item	Component		Corresponding Principal Component	Item	Component		Corresponding Principal Component
	1	2			1	2	
P-CAN1	0.443	-0.17	1	P-CAN26	0.467	0.257	1
P-CAN2	0.268	0.251	1	P-CAN27	-0.183	0.748	2
P-CAN3	-0.195	0.664	2	P-CAN28	0.583	-0.032	1
P-CAN4	0.533	-0.062	1	P-CAN29	0.612	0.064	1
P-CAN5	-0.23	0.614	2	P-CAN30	0.069	0.554	2
P-CAN6	0.638	-0.052	1	P-CAN31	-0.136	0.574	2
P-CAN7	-0.077	0.559	2	P-CAN32	-0.147	0.657	2
P-CAN8	0.54	0.143	1	P-CAN33	0.028	0.497	2
P-CAN9	0.021	0.592	2	P-CAN34	0.546	-0.088	1
P-CAN10	0.563	-0.265	1	P-CAN35	0.648	-0.048	1
P-CAN11	0.648	-0.152	1	P-CAN36	0.607	-0.066	1
P-CAN12	-0.072	0.546	2	P-CAN37	0.581	-0.101	1
P-CAN13	0.692	0.044	1	P-CAN38	0.052	0.556	2
P-CAN14	0.094	0.48	2	P-CAN39	0.594	-0.254	1
P-CAN15	0.613	-0.01	1	P-CAN40	-0.277	0.754	2
P-CAN16	0.55	-0.185	1	P-CAN41	0.713	-0.142	1
P-CAN17	0.543	0.204	1	P-CAN42	0.558	0.202	1
P-CAN18	0.257	0.397	2	P-CAN43	-0.046	0.594	2
P-CAN19	0.702	-0.01	1	P-CAN44	0.6	-0.096	1
P-CAN20	0.692	-0.018	1	P-CAN45	0.621	-0.296	1
P-CAN21	-0.005	0.532	2	P-CAN46	0.691	-0.097	1
P-CAN22	0.304	0.331	1	P-CAN47	-0.029	0.696	2
P-CAN23	0.687	0.166	1	P-CAN48	0.491	-0.265	1
P-CAN24	0.411	0.276	1	P-CAN49	-0.068	0.564	2
P-CAN25	0.505	-0.032	1	P-CAN50	-0.05	0.687	2

Note: The principal component (PC), denoted as P-CAN n, represents the nth item of the Pros and Cons of Anorexia Nervosa Scale (P-CAN). Given that P-CAN 22 exhibits similar loadings on two principal components, it has been assigned to PC 1 based on its theoretical alignment with the original scale. The extraction method used was PCA, with a rotation method of Varimax and Kaiser Normalization. The rotation converged in 3 iterations

Subsequently, a PCA was conducted on the 31 items of the Pro-AN subscale, with the extraction of six PCs specified according to the original scale’s structure (details in Table 6). The KMO value of 0.899, coupled with a highly significant Bartlett’s Test of Sphericity (χ^2^ = 3162.479, df = 465, *p* < 0.001), indicates a strong intercorrelation among variables and affirms the suitability for PCA. The Rotation Sums of Squared Loadings indicate that PC1 accounts for 13.753% of the total variance, PC2 accounts for 11.013%, PC3 for 10.098%, PC4 for 9.725%, PC5 for 8.448%, and PC6 for 7.493%, with a cumulative variance explanation of 60.531% across all six PCs. Among the six PCs, PC 2 corresponds to the Fertility/Sexuality subscale and PC 5 corresponds to the Communicate Emotions/Distress subscale of the original scale.

**Table 6:** Rotated component matrix for PCA results of the Pro-AN scales: six component solutions

Item	Component						Corresponding Principal Component
	1	2	3	4	5	6	
P-CAN1	0.24	0.356	-0.187	0.517	-0.039	0.158	4
P-CAN2	-0.037	0.039	0.087	0.17	0.675	-0.129	5
P-CAN4	0.105	0.085	0.483	0.503	0.255	-0.148	4
P-CAN6	0.281	0.107	0.119	0.729	0.218	0.071	4
P-CAN8	0.021	0.303	0.411	0.268	0.03	0.315	3
P-CAN10	0.159	0.356	0.377	0.434	-0.178	0.15	4
P-CAN11	0.346	0.172	0.132	0.64	0.059	0.166	4
P-CAN13	0.092	0.107	0.535	0.506	0.38	0.127	3
P-CAN15	0.399	-0.033	0.309	0.436	0.052	0.263	4
P-CAN16	0.222	0.78	0.094	0.112	0.053	0.065	2
P-CAN17	0.087	0.213	0.278	0.113	-0.006	0.744	6
P-CAN19	0.461	0.123	0.252	0.286	0.07	0.48	6
P-CAN20	0.392	0.245	0.068	0.428	0.161	0.391	4
P-CAN22	0.025	0.084	-0.03	-0.083	0.623	0.369	5
P-CAN23	0.251	0.098	0.547	0.147	0.357	0.325	3
P-CAN24	0.107	0.038	-0.057	0.121	0.221	0.744	6
P-CAN25	0.05	0.698	0.065	0.09	0.209	0.237	2
P-CAN26	0.086	0.137	0.222	0.046	0.763	0.083	5
P-CAN28	0.274	0.129	0.751	-0.01	0.154	0.078	3
P-CAN29	0.278	0.143	0.613	0.118	0.291	0.024	3
P-CAN34	0.17	0.767	0.143	0.106	0.131	0.023	2
P-CAN35	0.518	0.35	0.202	0.113	0.056	0.254	1
P-CAN36	0.449	0.023	0.326	0.405	-0.029	0.198	1
P-CAN37	0.251	0.806	0.168	0.097	-0.004	0.042	2
P-CAN39	0.709	0.165	0.257	0.054	0.057	0.011	1
P-CAN41	0.593	0.178	0.349	0.131	0.263	0.136	1
P-CAN42	0.179	0.049	0.334	0.106	0.644	0.199	5
P-CAN44	0.665	-0.037	0.175	0.298	0.007	0.157	1
P-CAN45	0.737	0.285	-0.01	0.256	-0.037	0.078	1
P-CAN46	0.579	0.251	0.362	0.162	0.136	0.067	1
P-CAN48	0.61	0.385	-0.148	0.229	0.066	-0.115	1

Note: The principal component (PC), denoted as P-CAN n, represents the nth item of the Pros and Cons of Anorexia Nervosa Scale (P-CAN). The extraction method utilized for this analysis was PCA, and the rotation method applied was Varimax with Kaiser Normalization. The rotation process converged after 10 iterations

Similarly, an identical analysis was performed on the 19 items associated with the Con-AN subscale, which identified four PCs (details in Table 7). The KMO value was 0.897, and Bartlett’s Test of Sphericity was significant (χ^2^ = 1666.307, df = 171, *p* < 0.001), confirming the appropriateness for a PCA. The four PCs explained 37.283%, 18.144%, 16.866%, and 16.076% of the variance respectively, amassing a cumulative explanation of 59.305%. In the four components of the Con-AN subscale, PC 2 corresponds to the Guilt subscale of the original measure, PC 3 corresponds to the Hatred while items 33 and 38 are absent. Trapped and Stifled do not clearly align with any specific PC.

**Table 7:** Rotated component matrix for PCA results of the Pro-AN scales: four component solutions

Item	Component				Corresponding Principal Component
	1	2	3	4	
P-CAN3	0.504	0.168	0.556	-0.08	3
P-CAN5	0.167	0.105	0.794	0.041	3
P-CAN7	0.029	0.703	0.332	-0.007	2
P-CAN9	0.317	0.616	0.178	-0.012	2
P-CAN12	0.082	0.134	0.728	0.115	3
P-CAN14	0.475	0.149	0.131	0.152	1
P-CAN18	0.094	0.029	0.126	0.85	4
P-CAN21	0.037	0.831	0.104	0.166	2
P-CAN27	0.422	0.232	0.63	0.171	3
P-CAN30	0.704	0.043	0.117	0.206	1
P-CAN31	0.795	0.141	0.126	-0.051	1
P-CAN32	0.611	0.244	0.314	0.115	1
P-CAN33	0.228	0.185	0.154	0.711	4
P-CAN38	0.503	0.088	0.273	0.251	1
P-CAN40	0.47	0.253	0.584	0.191	3
P-CAN43	0.244	0.146	0.58	0.19	3
P-CAN47	0.646	0.314	0.251	0.044	1
P-CAN49	0.274	0.765	0.012	0.03	2
P-CAN50	0.254	0.784	0.188	0.188	2

Note: The principal component (PC), P-CAN n represents the nth item of Pros and Cons of Anorexia Nervosa Scale. The extraction method used was Principal Component Analysis, and the rotation method applied was Varimax with Kaiser Normalization, which converged in 6 iterations

### Criterion-related validity

The correlation analysis revealed a significant negative association between Pro-AN and motivation, with a correlation coefficient of -0.233 (*p* < 0.01). Conversely, a significant positive correlation was observed between Con-AN and motivation, with a correlation coefficient of 0.499 (*p* < 0.001). Pro-AN and Con-AN showed significant correlation with the eating pathology (measured by EDE-Q), with Pro-AN at *r* = 0.279, *p* < 0.001 and Con-AN at *r* = 0.240, *p* < 0.001. The Con subscale of the P-CAN exhibited a significant negative correlation with BMI and the lowest BMI since the onset of the disorder, with a correlation coefficient of *r* = -0.214, *p* < 0.01 and − 0.273, *p* < 0.001, while Pro-AN did not show a significant correlation with BMI and the lowest BMI since the onset of the disorder.

## Discussion

The P-CAN scale is a questionnaire designed to quantitatively measure the positive and negative aspects of AN, based on themes identified through qualitative research [12], to enhance the understanding of patients’ experiences [13]. However, to the best of our knowledge, the P-CAN has only been utilized in Western contexts. Perceptions of mental disorders vary across different cultural settings, including experiences related to healthcare access, the quality of care received, and stigma [15]. These factors can significantly influence patients’ perceptions of the benefits and harms associated with their illness. China is witnessing a growing population of individuals with AN, making the management and treatment of eating disorders a matter of increasing concern [3, 4]. Considering the cultural differences in experiences of mental disorders [15] and the significant need in China [3], this study aims to validate the P-CAN scale among patients with AN in China.

Regarding content validity, experts in the relevant field in China have recognized the content of the P-CAN as appropriate based on the standards described in Almanasreh, Moles [28]. In terms of reliability, both the Pro and Con subscales of the P-CAN demonstrated high Cronbach’s alpha coefficients, indicating good internal consistency to the standards set described in Tavakol and Dennick [29]. However, the reliability coefficients for more specific dimensions were lower. This may be attributed to the limited number of items within these finer dimensions, with some containing as few as four items, which can affect consistency [30].

Regarding dimensionality, when divided into Pros and Cons, the Chinese version aligns almost perfectly with the original scale [12]. This indicates that both Chinese and Western groups share a fundamental agreement in their value judgments and negative consequences regarding AN. Item 29, “Anorexia helps me to control my emotions,” is the only item that exhibits a significant divergence between the Chinese context and the original scale [12]. The data from China supports the view that this statement is a benefit associated with the disorder, this perspective is also reflected in the content validation ratings provided by experts. In contrast, the original scale interprets it as a detriment, highlighting how AN can suppress emotional expression [12]. This difference may stem from the fact that, within Confucian culture, the control or concealment of emotions aligns with established principles [31], where being less emotional is regarded as favorable [32]. For Chinese participants, the notion that AN aids in emotional regulation is not perceived as a negative aspect.

However, a more granular breakdown into ten dimensions reveals significant differences between the Chinese context and the original scale [12]. In the context of a ten-factor PCA, there exists a degree of overlap among the items associated with these dimensions. Particularly for the original subscales ‘Stifled’ and ‘Trapped’, there are no corresponding principal components identified in the PCA. One plausible explanation is that the experiences of being stifled and trapped might not be differentiated among Chinese patients, as there tends to be a greater emphasis on sadness in emotional expression and feelings within this cultural framework [33]. Based on this, it is recommended that when using this scale in Chinese populations, greater consideration should be given to reporting the overall Pro-AN and Con-AN factors rather than more detailed dimensions and interpreting the results with greater nuance and caution at a more granular level.

In terms of criterion validity and sensitivity to clinical outcomes, through correlational analysis, it is evident that there exists a negative correlation between Pro-AN and motivation, while Con-AN and motivation exhibit a positive correlation, which is consistent with the original author’s construct [13]. Both Pro-AN and Con-AN show a positive correlation with eating pathology as measured by EDE-Q. This finding is consistent with the original study by Serpell, Teasdale [12] and aligns with the theoretical assumption that Pro-AN is closely linked to the maintenance of AN within the cognitive-interpersonal maintenance model proposed by Schmidt and Treasure [5]. However, no significant correlation was found between the Pro-AN and BMI and the lowest BMI since the onset of the disorder. Serpell, Teasdale [12] explained that BMI is not a reliable indicator of severity at a single point in time. Instead, changes in BMI or the average BMI over the past year may provide a more accurate reflection of the severity or chronic nature of the disorder [12]. While a significant correlation was observed between the Con-AN and BMI and the lowest BMI since the onset of the disorder among Chinese, indicating that perceived negative aspects of AN relate to BMI. Con-AN, as a consequence of the disorder, correlates with an increase in the severity of the illness, indicating a need for further exploration.

In clinical practice, accurately assessing a patient’s perspectives is crucial for tailoring effective treatment plans and improving therapeutic outcomes. Knowledge of factors associated with motivation to change is important to understand those who may have poorer treatment outcomes as well [7]. The P-CAN-C scale serves as a robust instrument tailored for the Chinese population, facilitating a comprehensive understanding of patients’ gains and obstacles throughout their illness trajectory. This tool enables the provision of precise interventions and simultaneously offers caregivers, therapists, and broader societal stakeholders a valuable lens through which to comprehend patients’ experiences.

### Limitations

The relatively small size of the AN population, with a prevalence of less than 0.1% in China [3], presents significant challenges in obtaining sufficient samples for research purposes. This limitation may affect the robustness of the statistical analyses conducted in this study. The sample characteristics may introduce selection bias: the predominant representation of partially recovered patients (as evidenced by BMI indices) limits the generalizability of findings to populations in an acute stage, while the very small number of male participants contributes to gender representation bias. The reliance on self-report measures introduces potential measurement inaccuracies, which may compromise data objectivity. The cross-sectional design fundamentally precludes causal inferences, particularly regarding the relationship between Pro and Con AN and eating pathology.

## Conclusions

The P-CAN-C demonstrates acceptable psychometric properties for assessing motivation-related factors in Chinese adults with AN. While the dimensions of Pro-AN and Con-AN exhibit cross-cultural consistency, the 10-subscale division show limited applicability within the Chinese cultural context. These findings suggest that clinicians and researchers should exercise caution when interpreting subscale scores and consider cultural nuances in their application.

### Future directions

Future research should prioritize qualitative investigations into the perspectives of Chinese patients with AN to further explore potential additional pros and cons across different cultural contexts. Building upon existing assessment tools, comparative studies examining the perspectives of both Eastern and Western patients with AN represent a promising area of inquiry. Additionally, given the increasing prevalence of AN among adolescents in China [4], there is an urgent need to adapt and validate these scales specifically for younger populations. The evaluation and reporting of the clinical application of the scale in China are also anticipated to be important directions for future research.

## Electronic supplementary material

Below is the link to the electronic supplementary material.

Supplementary Material 1

## Data Availability

The datasets generated and analyzed during the current study are available from the corresponding author on reasonable request.

## Abbreviations

AN: Anorexia Nervosa
P-CAN: Pros and Cons of Anorexia Nervosa Scale
P-CAN-C: Chinese Version of the Pros and Cons of Anorexia Nervosa Scale
Pro-AN: Positive Aspects of Anorexia Nervosa (valued nature)
Con-AN: Negative Aspects of Anorexia Nervosa (adverse consequences)
BMI: Body Mass Index
PCA: Principal Component Analysis
PCs: Principal Components
