# Supplementary material for: Psychometric properties of the Chinese version of the pros and cons of anorexia nervosa (P-CAN-C) scale: a validation study in patients with anorexia nervosa
Source: J Eat Disord. 2025 Jun 16;13:111. doi: 10.1186/s40337-025-01314-x (PMC12172320; doi:10.1186/s40337-025-01314-x)
Supplement: Supplementary file 1 — Supplementary Material 1 [file 40337_2025_1314_MOESM1_ESM.docx]

# Appendix

The translated Chinese version of the Pros and Cons of Anorexia Nervosa Scale (PCAN), as used in this study, is included in the Appendix for reference. This version was developed through a rigorous translation and cultural adaptation process to ensure its applicability to Chinese populations.

| 请阅读每个陈述，并在最能表达您对该陈述的同意或不赞同的列上打一个✓。这些问题没有对错之分，您的回答将完全保密，请尽量诚实地回答。  2=非常同意 1=较为赞同 0=无所谓 -1=较为不赞同 -2=非常不赞同 | | | | | |
| --- | --- | --- | --- | --- | --- |
| 1. 我觉得我的厌食症使我更健康了 | 2 | 1 | 0 | -1 | -2 |
| 2. 厌食症表达了我内心的痛苦 | 2 | 1 | 0 | -1 | -2 |
| 3. 厌食症使我无法感受生活 | 2 | 1 | 0 | -1 | -2 |
| 4. 厌食症帮助我应对生活 | 2 | 1 | 0 | -1 | -2 |
| 5. 厌食症使我感到沮丧 | 2 | 1 | 0 | -1 | -2 |
| 6. 我觉得因为厌食症，我对他人更有吸引力 | 2 | 1 | 0 | -1 | -2 |
| 7. 我为我的厌食症让他人担忧而感到难过 | 2 | 1 | 0 | -1 | -2 |
| 8. 我认为我的厌食症是可靠的和一贯的 | 2 | 1 | 0 | -1 | -2 |
| 9. 因为我的厌食症，我伤害了我身边的人 | 2 | 1 | 0 | -1 | -2 |
| 10. 厌食症使我的生活有条不紊 | 2 | 1 | 0 | -1 | -2 |
| 11. 厌食症使我超越他人 | 2 | 1 | 0 | -1 | -2 |
| 12. 我希望我的厌食症能够消失，让我一个人呆着! | 2 | 1 | 0 | -1 | -2 |
| 13. 我感到被我的厌食症所保护 | 2 | 1 | 0 | -1 | -2 |
| 14. 厌食症使我的情绪变得麻木 | 2 | 1 | 0 | -1 | -2 |
| 15. 患有厌食症意味着我可以穿我想穿的衣服 | 2 | 1 | 0 | -1 | -2 |
| 16. 厌食症使我能够避免月经的干扰 | 2 | 1 | 0 | -1 | -2 |
| 17. 厌食症是我擅长的事情 | 2 | 1 | 0 | -1 | -2 |
| 18. 我觉得自己无法摆脱厌食症的困扰 | 2 | 1 | 0 | -1 | -2 |
| 19. 厌食症表明我至少在某一方面比其他人做得更好 | 2 | 1 | 0 | -1 | -2 |
| 20. 厌食症是一种技能 | 2 | 1 | 0 | -1 | -2 |
| 21. 我讨厌因为我的厌食症而让父母担心 | 2 | 1 | 0 | -1 | -2 |
| 22. 我利用厌食症来向他人传达我的困扰/不快乐 | 2 | 1 | 0 | -1 | -2 |
| 23. 我的厌食症使我感到安全 | 2 | 1 | 0 | -1 | -2 |
| 24. 在厌食症上，我是一个专家 | 2 | 1 | 0 | -1 | -2 |
| 25. 因为有厌食症，我不必担心怀孕 | 2 | 1 | 0 | -1 | -2 |
| 26. 当进展不顺利时，厌食症是我的求救信号 | 2 | 1 | 0 | -1 | -2 |
| 27. 我讨厌厌食症控制我的生活的方式 | 2 | 1 | 0 | -1 | -2 |
| 28. 我的厌食症帮助我不失控 | 2 | 1 | 0 | -1 | -2 |
| 29. 厌食症帮助我控制自己的情绪 | 2 | 1 | 0 | -1 | -2 |
| 30. 厌食症占据了我所有的时间 | 2 | 1 | 0 | -1 | -2 |
| 31. 厌食症限制了我的情感表达 | 2 | 1 | 0 | -1 | -2 |
| 32. 厌食症让我不再是我 | 2 | 1 | 0 | -1 | -2 |
| 33. 我正在与我的厌食症作斗争 | 2 | 1 | 0 | -1 | -2 |
| 34. 厌食症终止了我的痛经 | 2 | 1 | 0 | -1 | -2 |
| 35. 因为厌食症，我可以推动我的身体超过以前的极限 | 2 | 1 | 0 | -1 | -2 |
| 36. 因为厌食症，我对自己的外表感觉更好了 | 2 | 1 | 0 | -1 | -2 |
| 37. 厌食症意味着我不再有经前综合征 | 2 | 1 | 0 | -1 | -2 |
| 38. 我已经厌倦了不断思考食物的问题 | 2 | 1 | 0 | -1 | -2 |
| 39. 厌食症使我的生活有了目标 | 2 | 1 | 0 | -1 | -2 |
| 40. 我讨厌厌食症 | 2 | 1 | 0 | -1 | -2 |
| 41. 我珍视我的厌食症，因为它让我感到安全 | 2 | 1 | 0 | -1 | -2 |
| 42. 我可以通过我的厌食症表达我的情绪 | 2 | 1 | 0 | -1 | -2 |
| 43. 我对厌食症感到餍足和疲劳 | 2 | 1 | 0 | -1 | -2 |
| 44. 我喜欢厌食症让我看起来的样子 | 2 | 1 | 0 | -1 | -2 |
| 45. 患有厌食症使我的身体更好地运作 | 2 | 1 | 0 | -1 | -2 |
| 46. 我的厌食症帮助我组织我的世界 | 2 | 1 | 0 | -1 | -2 |
| 47. 我觉得厌食症扼杀了我的自然情感 | 2 | 1 | 0 | -1 | -2 |
| 48. 由于我的厌食症，我的身体状况更好了。 | 2 | 1 | 0 | -1 | -2 |
| 49. 我为我的厌食症给我的朋友带来的担忧而感到内疚。 | 2 | 1 | 0 | -1 | -2 |
| 50. 我为厌食症对我的家庭造成的影响而感到遗憾。 | 2 | 1 | 0 | -1 | -2 |
